# Gold nanoparticles attenuates bacterial sepsis in cecal ligation and puncture mouse model through the induction of M2 macrophage polarization

**DOI:** 10.1186/s12866-018-1227-3

**Published:** 2018-08-17

**Authors:** Sujittra Taratummarat, Naunpun Sangphech, Chau Tran Bao Vu, Tanapat Palaga, Thunnicha Ondee, Saowapha Surawut, Amornpun Sereemaspun, Patcharee Ritprajak, Asada Leelahavanichkul

**Affiliations:** 1 Medical Microbiology, Interdisciplinary Program, Graduate School, Chulalongkorn University, Bangkok, Thailand; 2 Department of Microbiology, Faculty of Science, Chulalongkorn University, Bangkok, Thailand; 3 Oral Biology program, Faculty of Dentistry, Chulalongkorn University, Bangkok, Thailand; 4 Medical Sciences Program, Faculty of Medicine, Chulalongkorn University, Bangkok, Thailand; 5 Department of Anatomy, Faculty of Medicine, Chulalongkorn University, Bangkok, Thailand; 6 Department of Microbiology and Immunology and Research Unit of Oral Microbiology, Chulalongkorn University, Bangkok, Thailand; 7 Department of Microbiology, Faculty of Medicine, Chulalongkorn University, Bangkok, 10330 Thailand; 8 Center of Excellence in Immunology and Immune-mediated Diseases, Department of Microbiology, Faculty of Medicine Chulalongkorn University, Bangkok, Thailand

**Keywords:** Sepsis, Gold nanoparticles, Cecal ligation and puncture, Macrophage polarization

## Abstract

**Background:**

Gold nanoparticles (AuNP) have several biochemical advantageous properties especially for a candidate of drug carrier. However, the non-conjugated AuNP has a higher rate of cellular uptake than the conjugated ones. Spherical AuNP in a proper size (20–30 nm) is non-toxic to mice and shows anti-inflammatory properties. We tested if the administration of AuNP, as an adjuvant to antibiotics, could attenuate bacterial sepsis in cecal ligation and puncture (CLP) mouse model with antibiotic (imipenem/cilastatin).

**Results:**

Indeed, AuNP administration at the time of CLP improved the survival, blood bacterial burdens, kidney function, liver injury and inflammatory cytokines (TNF-α, IL-6, IL-1β and IL-10). AuNP also decreased M1 macrophages (CD86 + ve in F4/80 + ve cells) and increased M2 macrophages (CD206 + ve in F4/80 + ve cells) in the spleens of sepsis mice. The weak antibiotic effect of AuNP was demonstrated as the reduction of *E. coli* colony after 4 h incubation. In addition, AuNP altered cytokine production of bone-marrow-derived macrophages including reduced TNF-α, IL-6 and IL-1β but increased IL-10 at 6 and 24 h. Moreover, AuNP induced macrophage polarization toward anti-inflammatory responses (M2) as presented by increased *Arg1* (Arginase 1) and PPARγ with decreased *Nos2* (inducible nitric oxide synthase, iNos) and Nur77 at 3 h after incubation in vitro.

**Conclusions:**

The adjuvant therapy of AuNP, with a proper antibiotic, attenuated CLP-induced bacterial sepsis in mice, at least in part, through the antibiotic effect and the induction of macrophage function toward the anti-inflammatory responses.

## Background

Sepsis is a syndrome of organ dysfunction due to dysregulated host responses to systemic infection, independent of the type of organisms [1]. Bacterial sepsis is an important world-wide healthcare problem and it is a major cause of death in patients with clinically ill conditions [1]. The imbalance of pro- and anti-inflammatory immune responses is one of the important sepsis pathophysiology [2]. Both overt- and insufficient- immune responses to sepsis could be harmful to patients [2, 3]. As such, the moribund stage in sepsis could be a result of hyper-immune response or immune-suppressive reaction [4]. Hence, the rapid organism control with a proper immune modulation might be a proper strategy for sepsis attenuation.

Interestingly, the anti-inflammatory effect of gold derivatives is demonstrated and has been used as an anti-rheumatic drug [5, 6]. However, gold in these derivatives are in the active oxidizing forms [7] which might be improper to be used in the high oxidative stress condition of sepsis [8–10]. In contrast, gold nanoparticles (AuNP) are inert metallic form with the anti-inflammatory effect. AuNP attenuates macrophage pro-inflammatory cytokine production and spherical AuNP at the diameter of 20–30 nm is not toxic in either mice or adipose tissue macrophages [7]. AuNP in these diameters are suitable for targeting macrophages by both active and passive mechanisms [11]. It is also interesting to note that the properties and toxicities of AuNP depend on the differences in size and shape, surface modification and molecular conjugations [12, 13]. In addition, the facile production in different sizes and the molecular conjugations of AuNP is relatively un-complicate [7]. Hence, AuNP could be modified for several diverse applications [14, 15] and the conjugation of several active substances into AuNP has been studied [16]. However, un-conjugated AuNP demonstrates higher cellular uptake, at least in part, through the higher adsorption of serum proteins on the surface in comparison with the conjugated AuNP [17]. Thus, as a proof of concept for the initial study in sepsis, we selected to test with unconjugated-AuNP. Because macrophage is an important immune cell in sepsis and AuNP reduces pro-inflammatory cytokine production of macrophage, we hypothesize that the proper antibiotics in adjuvant with AuNP might attenuate sepsis severity. Then the effect of AuNP in sepsis was tested in a mouse model of cecal ligation and puncture (CLP) surgery with antibiotic administration. And we also explored if AuNP alter macrophage functions including cytokine production and macrophage polarization.

## Results

### Gold nanoparticles (AuNP) attenuate cecal ligation and puncture sepsis model

**Fig. 1:**
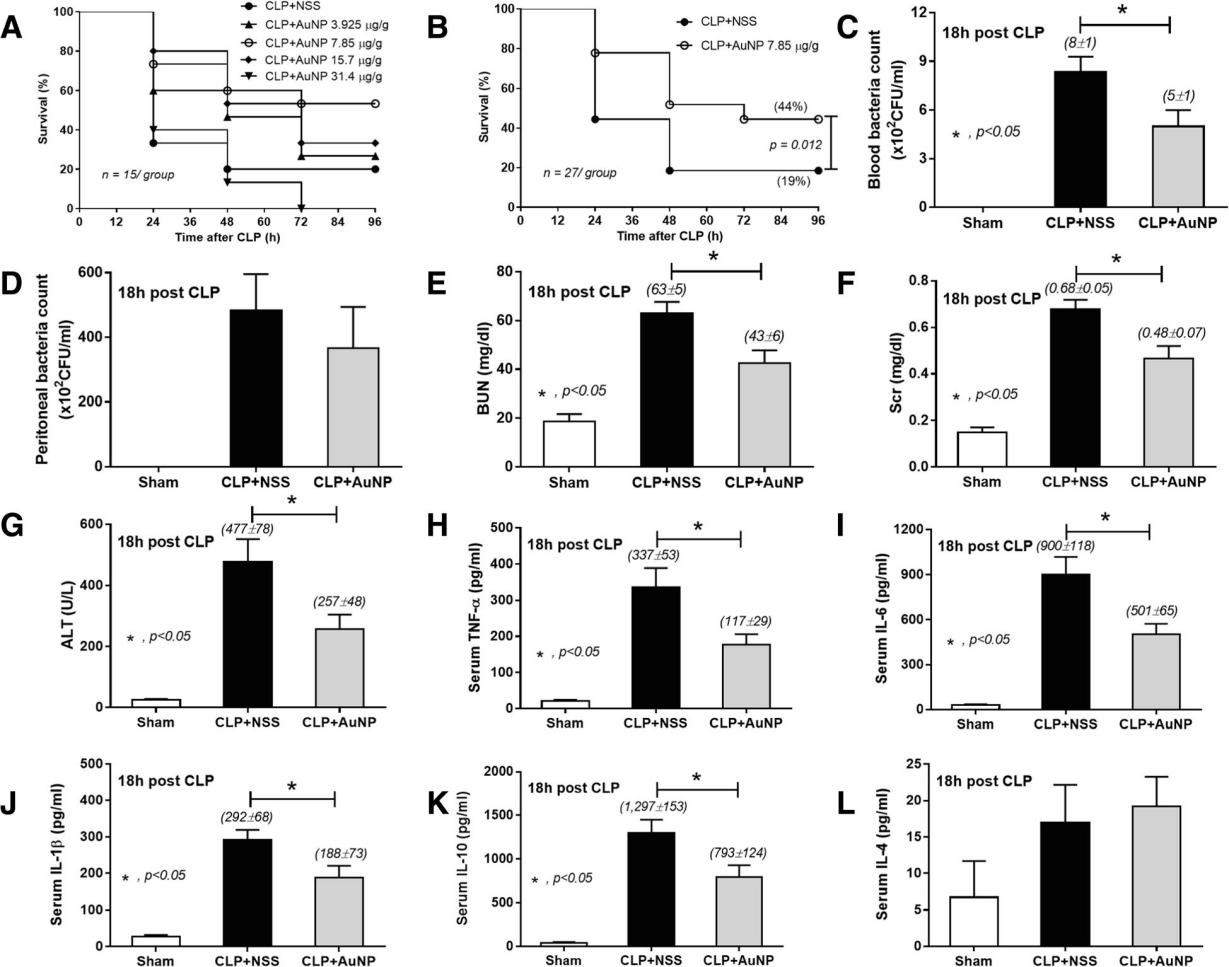
Gold nanoparticles (AuNP) in different doses were tested with a survival analysis (**a**). AuNP at 7.85 μg/gram body weight attenuated sepsis severity in cecal ligation and puncture (CLP) model. Survival analysis (**b**), bacterial burdens (in blood and peritoneal lavage) (**c**,  **d**), organ injury [blood urea nitrogen (BUN), serum creatinine (Scr), alanine transaminase (ALT)] (**e**-**g**) and serum cytokines (TNF-α, IL-6, IL-1β, IL-10 and IL-4) were demonstrated (**h**-**l**)

Due to the previous demonstration of anti-inflammatory effect of gold nanoparticles (AuNP) [7], we tested whether AuNP altered sepsis-induced mortality, renal dysfunction and liver injury in young outbred ICR mice treated with antibiotics. The anti-inflammatory effect of spherical AuNP in a diameter 21 nm at the dose of 7.85 μg/ gram body weight (approximately 200 ppm per mouse) is previously demonstrated in mice without any toxicities [7]. Hence, AuNP in this dose and at the lower and upper doses were tested with a survival analysis. Interestingly, only AuNP at 7.85 μg/g demonstrated a tendency for sepsis attenuation (Fig. 1a) despite a non-significant value difference. We, then, selected this dose to test further in the sepsis model. Indeed, AuNP that given immediately after CLP surgery attenuated sepsis mortality and reduced blood bacterial burdens, but not bacteria in peritoneal lavage (Fig. 1b-d). AuNP also improved kidney function [evaluated as blood urea nitrogen (BUN) and serum creatinine (Scr)] and liver injury [determined as alanine transaminase (ALT)] at 18 h post-CLP (Fig. 1e-g). Because cytokine response is one of the important factors in sepsis pathophysiology, pro- and anti- inflammatory cytokines were evaluated. Mice with AuNP treatment demonstrated the lower level of TNF-α, IL-6, IL-1β and IL-10, but not IL-4 (Fig. 1h-l).

**Fig. 2:**
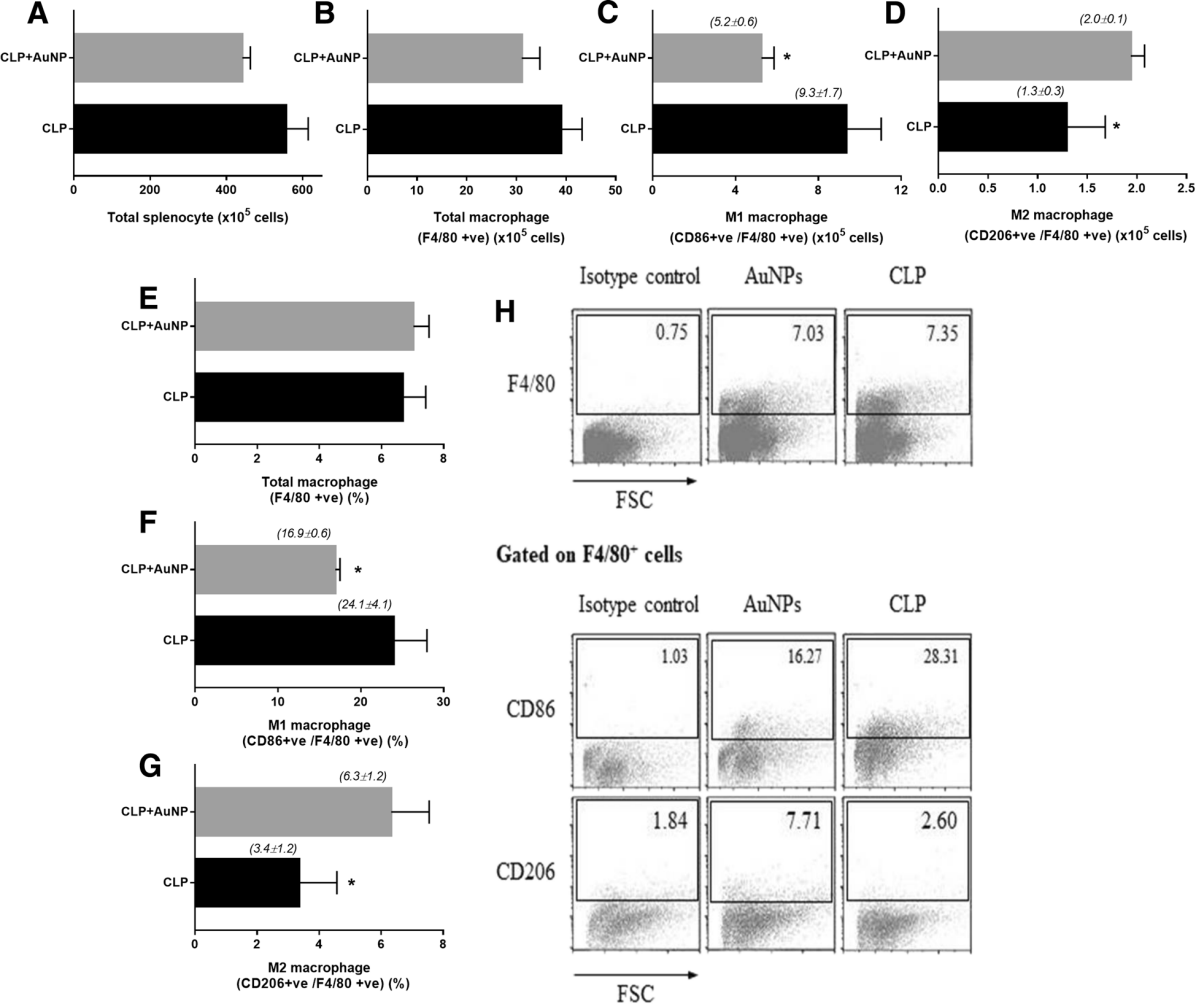
The quantitative flow-cytometric analysis of spleens from sepsis mice with and without gold nanoparticles (AuNP) administration in the total splenocyte (**a**), bulk macrophages (F4/80^+^; **b**), macrophages with M1 (CD86^+^ and F4/80^+^; **c**) and M2 (CD206^+^ and F4/80^+^; **d**) polarization and the percentage of the analysis (**e**-**g**) was demonstrated. The representatives of flow-cytometric analysis are shown (**h**). (*n* = 3–4/ group); *, *p* < 0.05

Because i) macrophage is important for cytokine activation in sepsis; ii) over pro-inflammatory cytokine production from macrophage leads to cytokine storm in sepsis [18]; and, iii) the balance of pro- (M1) and anti- inflammatory (M2) macrophage polarization might have an impact in sepsis [19], we explored the abundance of M1 and M2 macrophages in the spleens of sepsis mice. There was no difference in the percentage of macrophages (F4/80^+^) in both untreated and AuNP treated sepsis mice. However, AuNP treatment intriguingly showed the reduced percentage of pro-inflammatory macrophage (M1; F4/80^+^ and CD86^+^) and the increased percentage of anti-inflammatory macrophage (M2; F4/80^+^ and CD206^+^) in sepsis as demonstrated by the flow-cytometric analysis (Fig. 2).

### Anti-bacterial property and the induction of macrophages anti-inflammatory responses of gold nanoparticles (AuNP)

**Fig. 3:**
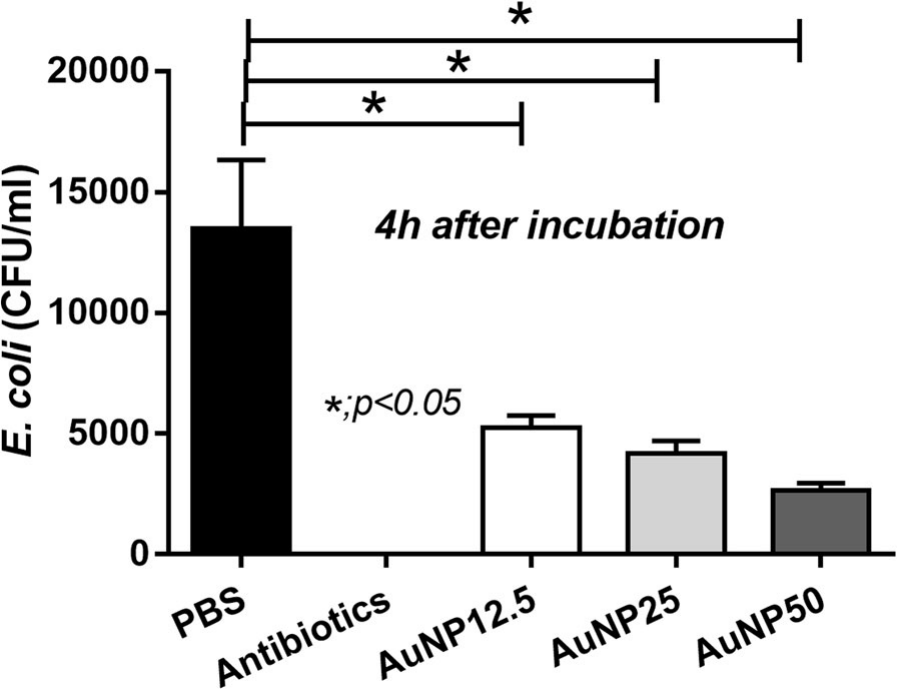
Anti-microbial activity of gold nanoparticles (AuNP) was demonstrated with the 4 h incubation with phosphate buffer solution (PBS), gentamicin antibiotic (antibiotic) and AuNP in different concentrations; 12.5, 25 and 50 ppm, respectively (triplicate independent experiments were performed)

**Fig. 4:**
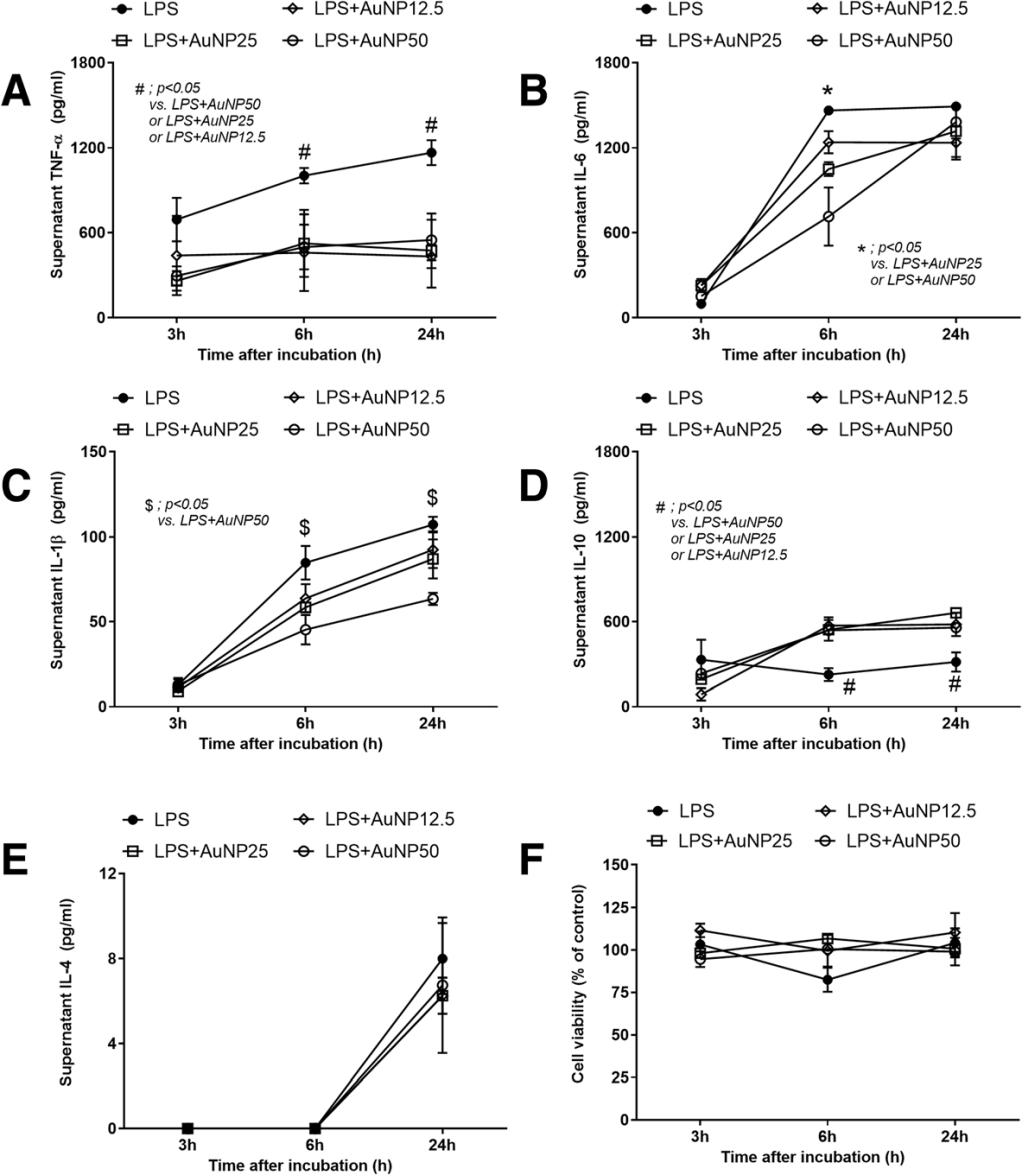
Macrophage cytokine production of TNF-α (**a**), IL-6 (**b**), IL-1β (**c**), IL-10 (**d**), IL-4 (**e**) and cell viability (**f**) after incubation with endotoxin (LPS) and gold nanoparticles (AuNP) in different concentrations; 12.5, 25 and 50 ppm, respectively, was demonstrated (triplicate independent experiments were performed)

Although cecal ligation and puncture (CLP) is a sepsis model with poly microbial organisms, gram-negative bacteremia (especially *E. coli*) is the predominant cause of death in CLP mice [20]. Hence, *E. coli* was selected to test in our experiments. Interestingly, AuNP demonstrated anti-microbial effect by the reduction of *E. coli* colonies after 4 h of the incubation (Fig. 3). Although the antibiotic effect of AuNP is weaker than gentamicin antibiotic, this, at least in part, supports the lower blood bacterial burdens in CLP mice with AuNP treatment (Fig. 1c). In addition, effect of AuNP on bone marrow derived macrophages was tested. As such, AuNP attenuated endotoxin-induced macrophage cytokine production as demonstrated by the lower supernatant TNF-α and IL-1β at 6 and 24 h of the incubation (Fig. 4a, c). AuNP reduced IL-6 only at 6 h but not at 24 h (Fig. 4b) and induced the higher IL-10, an anti-inflammatory cytokine, at 6 and 24 h of the incubation (Fig. 4d). However, AuNP did not have any effects on IL-4 (Fig. 4e). There was no AuNP dose-related effect on macrophage cytokine production on TNF-α, IL-1β and IL-10 at 6 and 24 h of the incubation. But there was a partially dose-related effect on IL-6 at 6 h of the incubation (Fig. 4). Hence, AuNP reduced several pro-inflammatory cytokines but demonstrated the different effects on anti-inflammatory cytokines (increased IL-10 and no effect on IL-4).

**Fig. 5:**
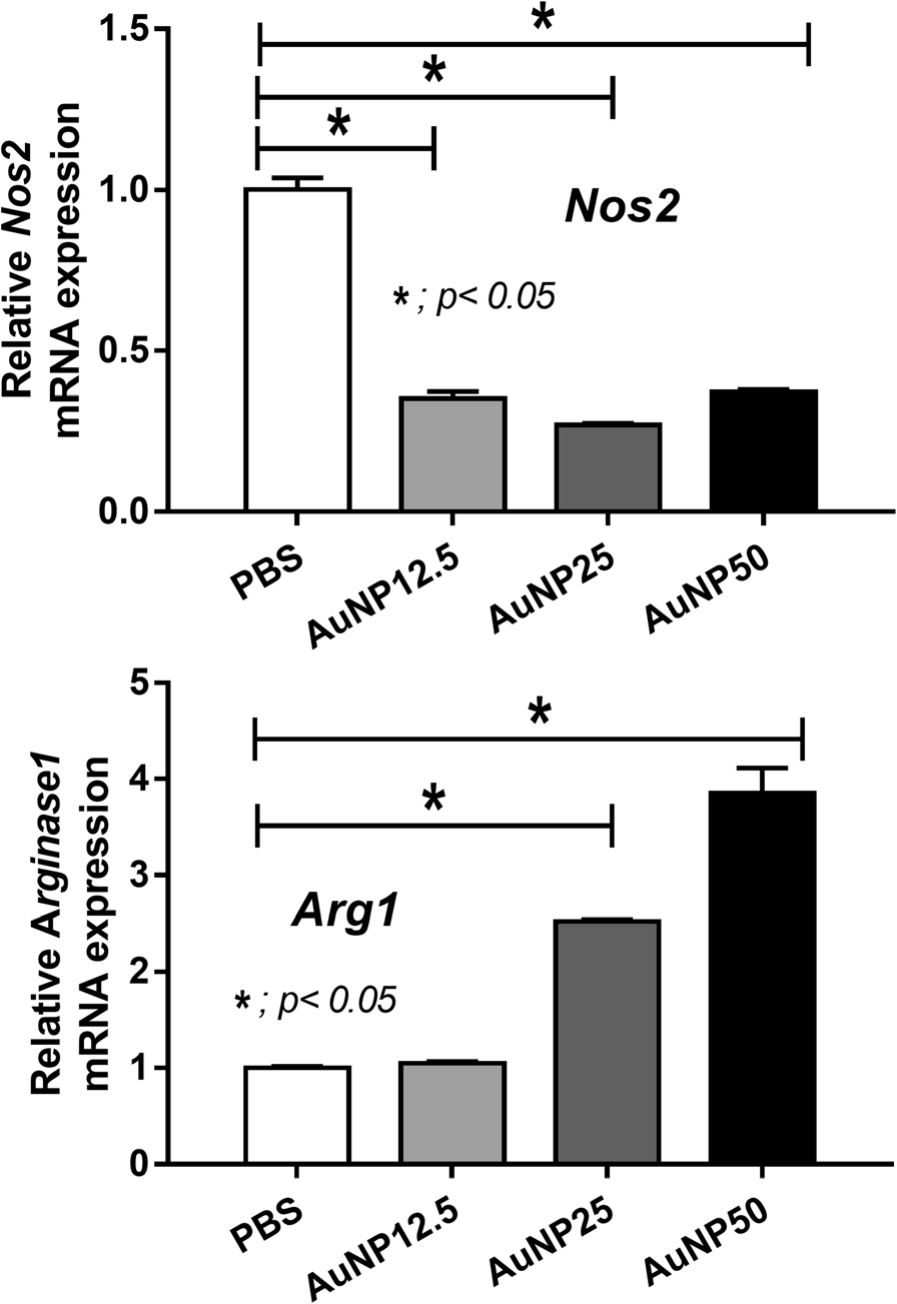
Macrophage polarization after activation by different concentrations of gold nanoparticles (AuNP), 12.5, 25 and 50 ppm, with control phosphate buffer solution (PBS) or endotoxin is shown. Relative mRNA expression in *iNOS* (**a**) and *Arginase1* (**b**) represents M1 and M2 macrophage polarization, respectively (duplicate independent experiments were performed)

**Fig. 6:**
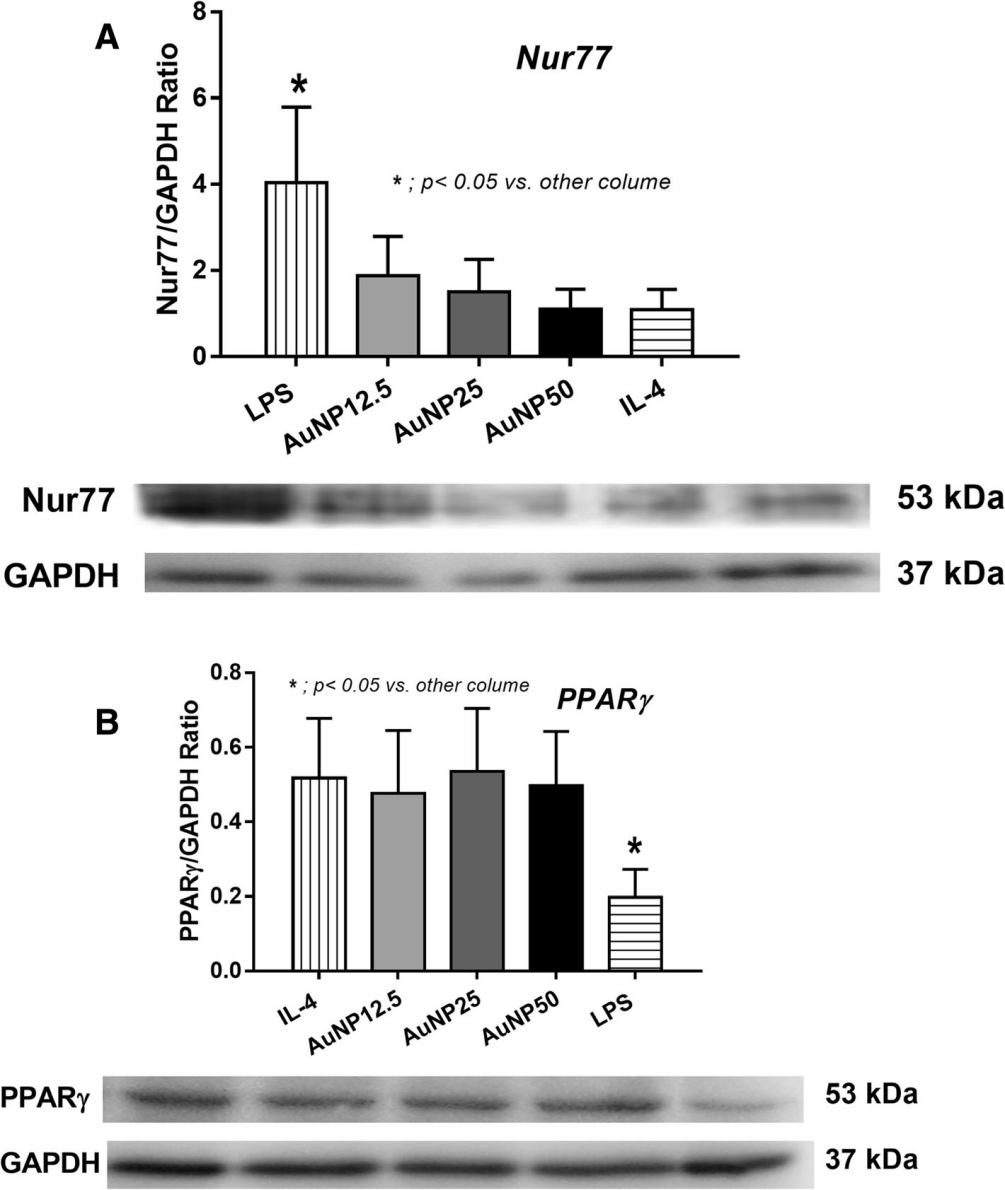
Macrophage polarization after activation by LPS (for M1 polarization), different concentrations of gold nanoparticles (AuNP; 12.5, 25 and 50 ppm) and IL-4 (for M2 polarization) were performed. The protein expression ratio by Western blot analysis of Nur77 (**a**) and PPARγ (**b**) represents M1 and M2 macrophage polarization, respectively, were demonstrated. And the representatives Western blot analysis are shown (triplicate independent experiments were performed)

Because high IL-10 production is one of the characteristics of anti-inflammatory macrophage (M2), we tested macrophage polarization with mRNA and protein expression. Indeed, the incubation of macrophage with AuNP reduced macrophage production of inducible nitric oxide synthase (*iNOS*) and increased *Arginase 1* (Fig. 5). M1 activation property of AuNP was lower than LPS, a potent M1 polarization stimulator, and not different from IL-4, a negative control of M1 polarization stimulator, as determined by the abundance of Nur77 (Fig. 6a). On the other hand, M2 activation property of AuNP was not different from IL-4, a potent M2 polarization stimulator, as demonstrated by the non-different PPARγ activation (Fig. 6b). However, there was no dose-related characteristic in these properties. These data demonstrated the influence of AuNP on the anti-inflammatory functions of macrophages.

## Discussion

The adjuvant effect of spherical gold nanoparticles (AuNP) on antibiotic for the attenuation of bacterial sepsis was demonstrated in a mouse model of polymicrobial sepsis with cecal ligation and puncture (CLP) surgery. AuNP administration, with an appropriate antibiotic, improved sepsis survival through the alteration of macrophage function toward the anti-inflammatory direction.

### AuNP attenuated polymicrobial sepsis

While AuNP in a small diameter are toxic to cells [21], AuNP in a proper range of diameters (20–30 nm) induce the anti-inflammatory effect [7, 22–24]. In addition, the intravenous administration shows the lowest toxicity in comparison with either oral or intra-peritoneal route [25]. Despite the relatively easy conjugation process of AuNP, un-conjugated AuNP shows higher cellular uptake property [17]. Because sepsis is an imbalance between pro- and anti- inflammation [1] and CLP is a sepsis model of hyperinflammation [26], AuNP might be beneficial in this model. Hence, we administered AuNP at 21 nm diameter through the tail vein at post-CLP surgery together with subcutaneously antibiotic administration. Only AuNP at 7.85 μg/g, but not the lower and the higher doses, showed a tendency of reduced sepsis mortality rate. The mortality rate of high-dose AuNP, compared with non-treated CLP, was also demonstrated, indicating a possible dose-related toxicity. However, with a proper dose, AuNP attenuated sepsis as measured by mortality, renal dysfunction, liver injury and cytokine levels. Additionally, we also demonstrated a weak antibiotic effect of AuNP supporting a previous publication [27]. The AuNP antibiotic effect might, at least in part, be responsible for the lower blood bacterial burdens.

### Anti-inflammatory effect of AuNP

Due to the rapid natural history of CLP sepsis, the role of macrophage, an important innate immune cells, should be predominant. Interestingly, macrophage polarization is one of the host mechanism to control the proper direction of immune responses; M1 for the pro- and M2 for the anti- inflammatory direction [28]. Indeed, we demonstrated that AuNP decreased pro-inflammatory macrophage (M1) and increased anti-inflammatory macrophage (M2) both in vitro and in vivo, supporting anti-inflammatory effect of AuNP in a previous study [7]. AuNP induced high-*Arginase 1* and low-*iNOS*, the biomarker of M2 and M1 polarization, respectively [29]. Because the predominant IL-10 production is one of the properties of M2 macrophage [28], we tested cytokine respones. As such, AuNP reduced pro-inflammatory cytokines (TNF-α, IL-6 and IL-1β) but increased anti-inflammatory cytokine (IL-10) in vitro.

It is interesting to note that serum IL-10 in CLP mice with AuNP treatment is not higher than CLP in the control group. This might due to the diversity of AuNP responses in the different cell types. While AuNP predominantly attenuates IL-10 from macrophages, it is possible that AuNP has no effect on other IL-10 producing cells (eg. non-immune cell and lymphocyte) [22]. The response of other cells against AuNP is interesting but out of the scope of our study. On the other hand, the lower serum IL-10 after AuNP treatment might be a balance immune response to the decreased pro-inflammatory cytokines [23]. In addition, AuNP did not show any effects on IL-4, another anti-inflammatory cytokine, both in vivo and in vitro. IL-4 is a well-known predominant cytokine of Th2 cell (immune cell of adaptive immunity) with a lesser influence in innate immune response in sepsis [30, 31]. Hence, the non-effective IL-4 attenuation of AuNP in our CLP mice implied the lesser impact of AuNP against Th2 in comparison with macrophage [24]. Further studies on this topic will be of great interest.

Although the mechanisms of AuNP-induced M2 polarization could not be concluded, nanoparticle size and arginine-binding (−modification) property might be important. As such, the binding activity of metal nanoparticle against amino acid and anti-inflammation of copper nano-particles through arginase modification are mentioned [32]. Perhaps, the proper size of AuNP allows a proper uptake [12, 33], enhances AuNP-arginine binding [34], induces cell stress [35] and arginase modifications leads to M2 polarization. And M2 polarization attenuates polymicrobial sepsis as previously mentioned [36]. More studies are needed to prove this hypothesis. Despite the lack of mechanistic details, our results support the role of AuNP and macrophage manipulation against bacterial sepsis.

## Conclusions

We demonstrated an adjuvant effect of spherical AuNP in 21 nm diameter on polymicrobial sepsis mouse model. This therapeutic effect was, at least in part, responsible from the induction of macrophage toward anti-inflammatory responses. Although the un-conjugated AuNP was used in our study, the conjugated AuNP with the proper agents might be more beneficial.

## Methods

### Gold nanoparticle preparation

Gold nanoparticles were synthesized following the standard citrate-reduction method as previously described [7, 16]. In brief, HAuCl_4_.3H_2_0 (1 mM) in Milli-Q water with 38.8 mM of sodium citrate was heated at 90 °C. The temperature altered the color of the solution from yellow into deep blue, and shortly afterwards, to deep red. Then the solution in deep red color was heating for another 15 min followed by continuous stirring in room temperature for another 15 min. The final solution containing spherical gold nanoparticles (AuNP) with average size of 21.3 ± 0.7 nm was stored at 4 °C, prevented from light until use. Size was confirmed by Ultra-Violet-Visible spectroscopy (Beckman Coulter DU 800 Spectrophotometer, Brea, CA, USA) (data not shown).

### Animals and animal models

The animal procedures followed the US National Institutes of Health (NIH) animal care and use protocols (#85–23, revised 1985) were implimented. Only male, 8-weeks-old, ICR mice (National Laboratory Animal Center, Nakornpathom, Thailand) were used to avoid the gender difference in sepsis severity [37]. The animal protocols have been approved by the Institutional Animal Care and Use Committee of the Faculty of Medicine, Chulalongkorn University, Bangkok, Thailand.

Cecal ligation and puncture (CLP) procedures followed the previous publications [30]. Briefly, 10 mm from cecal tip of the large bowel was ligated with silk 2–0, punctured twice with a 21-gauge needle through an abdominal incision under isoflurane anesthesia. Tramadol (an analgesic drug) at 10 mg/kg in 0.2 ml of normal saline solution (NSS), for analgesia and imipenem/cilastatin (an antibiotic) at 14 mg/kg in 0.2 ml of NSS] was administered intraperitoneally and subcutaneously, respectively, at post-operation and at 6 h later. AuNP diluted in 0.2 ml of NSS were administered through the tail vein at post-operation. AuNP at 3.925, 7.85, 15.7 and 31.4 μg/ gram body weight were tested with survival experiments in CLP mice and the effective dose (7.85 μg/ g) were used in other experiments. Blood was collected through cardiac puncture under isoflurane anesthesia at 18 h-post CLP. Whole blood was used for bacterial burden analysis and the rest of blood was centrifuged at 13,000 g for 5 min to separate serum and kept at − 80 °C until analysis. In the survival study, tramadol and antibiotic were administered once daily for 3 days and until 5 days of observation, respectively.

### Mouse blood sample analysis

As for quantitative bacterial analysis of blood and peritoneal cavity, 25 μl of blood and 1 μl of peritoneal fluid from the right para-mesenteric recess, respectively, were spread directly onto blood agar plates (Oxoid, Hampshire, UK), kept at 37 °C under aerobic conditions, and bacterial colonies were enumerated at 24 h. Serum creatinine (Scr), blood urea nitrogen (BUN) and alanine transaminase (ALT) was measured with QuantiChrom Creatinine Assay (DICT-500; Bioassay, Hayward, CA, USA), QuantiChrome Urea assay (DIUR-500; Bioassay) and EnzyChrom ALT assay (EALT-100, BioAssay). Serum cytokines (TNF-α, IL-6, IL-1β, IL-10 and IL-4) were measured with ELISA (eBioscience, San Diego, CA, USA). All assays were performed according to the manufacturer’s protocol.

### Flow-cytometric analysis of spleen macrophage

Flow-cytometric analysis was performed following the standard protocol. In brief, spleens were minced in supplemented RPMI-1640 (Roswell Park Memorial Institute media), and the cells were centrifuged at 300 g for 5 min at 4 °C. Red blood cells were removed using lysis buffer (Biolegend, CA, USA) and the splenocytes were washed twice in supplemented RPMI-1640. The cells were passed through a cell strainer before cell counting. Subsequently, the splenocytes were block with Fc block (anti-mouse CD16/32 mAbs; Biolegend); then were stained with the fluorochrome-conjugated antibodies against mouse F4/80 and CD86 (Biolegend). For intracellular staining, after Fc blocking and F4/80 surface staining, the cells were fixed in 4% paraformaldehyde overnight. Next, the cells were washed in the intracellular staining buffer and stained with fluorochrome-conjugated antibodies against mouse CD206 (Biolegend). Isotype-matched controls were used as a negative staining. All stained cells were analyzed by flow cytometry (Cytoflex, Beckman Coulter, IN, USA) and the data were analyzed using Kaluza software (Beckman Coulter).

### Antibiotics activity of gold nanoparticles

The procedure for determining antibiotic activity followed a previous publication [27]. *Escherichia coli* ATCC 25922 (ATCC, Manassas, VA, USA) was sub-cultured in Tryptic soy broth (Thermo Scientific, Waltham, MA, USA) at 37 °C for 12 h. *E. coli* approximately at 2.4 × 10^6^ CFU, as determined by a spectrophotometer (ELx808 absorbance reader; BioTek, Shoreline, WA) at optical density 600 nm at 0.003 (OD 600 nm at 0.003), were diluted in Tryptic soy broth (Thermo Scientific) and added with AuNP in different concentrations: 12.5, 25 and 50 ppm, respectively. Then the solutions were incubated at 37 °C for 4 h. After that, the supernatant in serial dilution was plated in Tryptic soy agar (Thermo Scientific), kept at 37 °C overnight before bacterial colony enumeration. *E. coli* in Tryptic soy broth alone or in 100 μg/ml of gentamicin were used as the positive and negative control group, respectively.

### Bone marrow derived macrophage preparation

Macrophages were derived from bone marrow (BM) following the previous published methods [3, 31] and bone marrow from femurs were obtained following the previous published methods [38]. In brief, BM cells were obtained from femurs by sacrificed mice under euthanize condition and open the abdominal cavity to find the pelvic-hip joint. Then cut off the hind leg above the pelvic-hip joint and cut off the tibia at below the knee joint. Remove the muscles and tissues around the femur with sterile forceps, scissors and gauze. After that, centrifuge at 6000 rpm for 4 °C to collect cells from bone marrow and incubated for 7 days with Dulbecco’s Modified Eagle Medium (DMEM) supplemented with 10% fetal bovine serum (FBS), 1% penicillin/streptomycin, 4-(2-hydroxyethyl)-1-piperazineethanesulfonic acid (HEPES) with sodium pyruvate in a humidified 5% CO_2_ incubator at 37 °C. Conditioned media of the L929 cell line, containing macrophage-colony stimulating factor, at 20% *w*/*v* were used to induce macrophages from BM-derived pleuripotent stem cells. Cells were harvested with cold phosphate buffer solution (PBS) and the macrophage phenotype was analyzed with anti-F4/80 and anti-CD11c antibody staining (BioLegend, San Diego, CA, USA) by flow cytometry (data not shown).

### Induction of macrophage cytokine production protocol

BM-derived macrophages at 1 × 10^5^ cells/well were plated in 96-well tissue culture plate, then incubated with 100 ng/ml of LPS of *Escherichia coli* 026:B6 (Sigma-Aldrich, St. Louis, MO, USA) and different concentration of AuNP: 12.5, 25 and 50 ppm, respectively, in 5% CO_2_ incubator at 37 °C for 3, 6 and 24 h. AuNP was adjusted into the similar volume for the incubation. At each time point, the culture supernatant was collected and stored at -80 °C until analysis. Then the supernatant was measured for cytokines (TNF-α, IL-6, IL-1β, IL-10 and IL-4) with ELISAs assay (eBioscience, San Diego, CA, USA) followed the manufacturer’s instructions. In addition, macrophage viability was measured after the incubation by the MTS assay (One Solution Cell Proliferation Assay, Promega Corporation, Madison, Wis, USA) according to the manufacturer’s instructions. Cell viability at each time point in the different conditions was calculated into percentage of cell viability with PBS control.

### Analysis of macrophage polarization

The protocol has been adapted from a previously published method [29, 32]. In brief, BM-derived macrophages 5 × 10^5^ cells/well were plated in 12-well tissue culture plate. Cells were activated into M1 and M2 with 100 ng/ml of LPS (Sigma-Aldrich) and 10 ng/ml of IL-4 (Sigma-Aldrich), respectively, with the presentation of AuNP in the different concentrations (12.5, 25 and 50 ppm) or PBS control. All groups were incubated in 5% CO_2_ incubator at 37 °C for 3 h, then the culture supernatant was removed and 400 ul of TRIzol reagent (Invitrogen, Carlsbad, CA, USA) was added per well to extract RNA from the macrophages followed to the manufacturer’s protocol. The purified RNA was synthesized into complementary DNA, amplified by quantitative polymerase chain reaction (qPCR) by used of *iNOS* and *Arginase1* for characterize of M1 and M2 macrophages, respectively. The nucleotide sequences were as followed: *iNOS,* forward, 5’-CCCTTCCGAAGTTTCTGGCAGCAGC-3′,reverse, 5’-GGCTGTCAGAGCCTCGTGGCTTT G-3′; *Arginase1,* forward, 5’-CAGAAGAATGGAAGAGTCAG-3′,reverse: 5’-CAGATATGCA GGGAGTCACC-3′. The expression of each gene was normalized to the expression of β-actin by the 2 − ΔΔCT method.

In addition, macrophage polarization was supported by Western blot analysis of Nur77 and PPARγ for M1 and M2 characterizations, respectively. In brief, macrophages 1 × 10^6^ cells/well were plated in a 12-well tissue culture plate with the conditions of LPS, IL-4 and AuNP. The cell harvest procedures were performed as mentioned above. Then the cells were added with RIPA lysis and extraction buffer, sonicated, centrifuged 10 min at 1500 rpm, 4 °C for the supernatant collection. Protein concentrations were determined by the bicinchoninic acid (BCA) assay and 20 μg proteins were separated by SDS-PAGE and transfer to Polyvinylidene difluoride (PVDF) membrane (activated by 100% methanol). The membrane was blocked with 5% bovine serum albumin (BSA) in tris-buffered saline with tween® 20 solution, then incubated at 4 °C overnight with anti-Nur77 (from Barbara Osborne, MA, USA) or anti-PPARγ (Cell Signaling Technology, Denvers, MA, USA) and anti-GAPDH (Santa Cruz Biotechnology, Dallas, TX, USA). The chemiluminescence bands were detected by the chemiluminescence detector (ImageQuant LAS 4000, GE Healthcare Life Sciences, Chicago, IL, USA).

### Statistical analysis

Data were analyzed as mean ± standard error (SE); the differences between groups were examined for statistical significance by one-way analysis of variance (ANOVA) followed by Bonferroni analysis for the experiments with multiple time-point data collection. The survival analysis was determined by log-rank test. The repeated measures analysis of variance (ANOVA) with Bonferroni post hoc analysis was used for the analysis of the time-course experiments. All statistical analyses were performed with SPSS 11.5 software (SPSS, IL, USA). *P* value < 0.05 was considered statistically significant.

## Abbreviations

ALT: Alanine transaminase
ANOVA: Analysis of variance
AuNP: Gold nanoparticles
BCA: Bicinchoninic acid
BM: Bone marrow
BSA: Bovine serum albumin
BUN: Blood urea nitrogen
CLP: Cecal ligation and puncture
DMEM: Dulbecco’s Modified Eagle Medium
ELISA: Enzyme linked immunosorbent assays
FBS: Fetal bovine serum
HEPES: 4-(2-hydroxyethyl)-1-piperazineethanesulfonic acid
IL-10: Interleukin 10
IL-1β: Interleukin 1 beta
IL-4: Interleukin 4
IL-6: Interleukin 6
*iNOS*: inducible nitric oxide synthase
NSS: Normal saline solution
PBS: Phosphate buffer solution
PVDF: Polyvinylidene difluoride
qPCR: Quantitative polymerase chain reaction
Scr: Serum creatinine
SE: Standard error
TNF-α: Tumor necrosis factor-alpha

## References

[1] Singer M, Deutschman CS, Seymour CW, Shankar-Hari M, Annane D, Bauer M (2016). The third international consensus definitions for Sepsis and septic shock (Sepsis-3). JAMA.

[2] Doi K, Leelahavanichkul A, Yuen PST, Star RA (2009). Animal models of sepsis and sepsis-induced kidney injury. J Clin Invest.

[3] Ondee T, Surawut S, Taratummarat S, Hirankan N, Palaga T, Pisitkun P, et al. FC Gamma Receptor IIB Deficient Mice: A Lupus Model with Increased Endotoxin Tolerance-Related Sepsis Susceptibility. Shock. 2017;47(6):743-752. 10.1097/SHK.0000000000000796 27849678

[4] Hotchkiss RS, Monneret G, Payen D (2013). Immunosuppression in sepsis: a novel understanding of the disorder and a new therapeutic approach. Lancet Infect Dis.

[5] Kean WF, Kean IRL (2008). Clinical pharmacology of gold. Inflammopharmacology.

[6] Ujfalussy I, Koó É, Seszták M, Gergely P (2003). Termination of disease-modifying antirheumatic drugs in rheumatoid arthritis and in psoriatic arthritis. Z Rheumatol.

[7] Chen H, Dorrigan A, Saad S, Hare DJ, Cortie MB, Valenzuela SM (2013). In vivo study of spherical gold nanoparticles: inflammatory effects and distribution in mice. PLoS One.

[8] Alobaidi R, Basu RK, Goldstein SL, Bagshaw SM (2015). Sepsis-associated acute kidney injury. Semin Nephrol.

[9] Arulkumaran N, Deutschman CS, Pinsky MR, Zuckerbraun B, Schumacker PT, Gomez H (2016). MITOCHONDRIAL FUNCTION IN SEPSIS. Shock (Augusta, Ga).

[10] Draganov D, Teiber J, Watson C, Bisgaier C, Nemzek J, Remick D (2010). PON1 and oxidative stress in human sepsis and an animal model of sepsis. Adv Exp Med Biol.

[11] Pissuwan D, Valenzuela SM, Killingsworth MC, Xu X, Cortie MB (2007). Targeted destruction of murine macrophage cells with bioconjugated gold nanorods. J Nanopart Res.

[12] Chithrani BD, Ghazani AA, Chan WC (2006). Determining the size and shape dependence of gold nanoparticle uptake into mammalian cells. Nano Lett.

[13] Fischer HC, Chan WC (2007). Nanotoxicity: the growing need for in vivo study. Curr Opin Biotechnol.

[14] Dykman LA, Khlebtsov NG (2011). Gold nanoparticles in biology and medicine: recent advances and prospects. Acta Nat.

[15] Mieszawska AJ, Mulder WJM, Fayad ZA, Cormode DP (2013). Multifunctional gold nanoparticles for diagnosis and therapy of disease. Mol Pharm.

[16] Pissuwan D, Cortie C, Valenzuela S, Cortie M (2007). Gold nanosphere-antibody conjugates for hyperthermal therapeutic applications. Gold Bull.

[17] Chithrani BD, Chan WC (2007). Elucidating the mechanism of cellular uptake and removal of protein-coated gold nanoparticles of different sizes and shapes. Nano Lett.

[18] Cavaillon JM, Adib-Conquy M (2005). Monocytes/macrophages and sepsis. Crit Care Med.

[19] Ka MB, Daumas A, Textoris J, Mege JL (2014). Phenotypic diversity and emerging new tools to study macrophage activation in bacterial infectious diseases. Front Immunol.

[20] Hyde SR, Stith RD, McCallum RE (1990). Mortality and bacteriology of sepsis following cecal ligation and puncture in aged mice. Infect Immun.

[21] Brandenberger C, Rothen-Rutishauser B, Mühlfeld C, Schmid O, Ferron GA, Maier KL (2010). Effects and uptake of gold nanoparticles deposited at the air–liquid interface of a human epithelial airway model. Toxicol Appl Pharmacol.

[22] Shaw DM, Merien F, Braakhuis A, Dulson D. T-cells and their cytokine production: the anti-inflammatory and immunosuppressive effects of strenuous exercise. Cytokine. 2017; 10.1016/j.cyto.2017.10.001 29021092

[23] Hotchkiss RS, Monneret G, Payen D (2013). Sepsis-induced immunosuppression: from cellular dysfunctions to immunotherapy. Nat Rev Immunol.

[24] Edholm ES, Rhoo KH, Robert J (2017). Evolutionary aspects of macrophages polarization. Results Probl Cell Differ.

[25] Zhang XD, Wu HY, Wu D, Wang YY, Chang JH, Zhai ZB (2010). Toxicologic effects of gold nanoparticles in vivo by different administration routes. Int J Nanomedicine.

[26] Doi K, Hu X, Yuen PST, Leelahavanichkul A, Yasuda H, Kim SM (2008). AP214, an analogue of α-melanocyte-stimulating hormone, ameliorates sepsis-induced acute kidney injury and mortality. Kidney Int.

[27] Zhou Y, Kong Y, Kundu S, Cirillo JD, Liang H (2012). Antibacterial activities of gold and silver nanoparticles against Escherichia coli and bacillus Calmette-Guerin. J Nanobiotechnology.

[28] Kimura T, Nada S, Takegahara N, Okuno T, Nojima S, Kang S (2016). Polarization of M2 macrophages requires Lamtor1 that integrates cytokine and amino-acid signals. Nat Commun.

[29] Jablonski KA, Amici SA, Webb LM, JdD R-R, Popovich PG, Partida-Sanchez S (2015). Novel Markers to Delineate Murine M1 and M2 Macrophages. PLOS ONE.

[30] Leelahavanichkul A, Souza ACP, Street JM, Hsu V, Tsuji T, Doi K (2014). Comparison of serum creatinine and serum cystatin C as biomarkers to detect sepsis-induced acute kidney injury and to predict mortality in CD-1 mice. Am J Physiol Ren Physiol.

[31] Surawut S, Ondee T, Taratummarat S, Palaga T, Pisitkun P, Chindamporn A (2017). The role of macrophages in the susceptibility of fc gamma receptor IIb deficient mice to Cryptococcus neoformans. Sci Rep.

[32] Wongchana W, Lawlor RG, Osborne BA, Palaga T (2015). Impact of Notch1 deletion in macrophages on Proinflammatory cytokine production and the outcome of experimental autoimmune encephalomyelitis. J Immunol.

[33] Albanese A, Tang PS, Chan WC (2012). The effect of nanoparticle size, shape, and surface chemistry on biological systems. Annu Rev Biomed Eng.

[34] Tomoaia G, Frangopol PT, Horovitz O, Bobos LD, Mocanu A, Tomoaia-Cotisel M (2011). The effect of arginine on gold nanoparticles in colloidal solutions and in thin films. J Nanosci Nanotechnol.

[35] Shan B, Wang X, Wu Y, Xu C, Xia Z, Dai J (2017). The metabolic ER stress sensor IRE1alpha suppresses alternative activation of macrophages and impairs energy expenditure in obesity. Nat Immunol.

[36] Nemeth K, Leelahavanichkul A, Yuen PS, Mayer B, Parmelee A, Doi K (2009). Bone marrow stromal cells attenuate sepsis via prostaglandin E(2)-dependent reprogramming of host macrophages to increase their interleukin-10 production. Nat Med.

[37] Angele MK, Pratschke S, Hubbard WJ, Chaudry IH (2014). Gender differences in sepsis: cardiovascular and immunological aspects. Virulence.

[38] Liu X, Quan N (2015). Immune cell isolation from mouse femur bone marrow. Bio-protocol.

